# P311 Deficiency Leads to Attenuated Angiogenesis in Cutaneous Wound Healing

**DOI:** 10.3389/fphys.2017.01004

**Published:** 2017-12-06

**Authors:** Song Wang, Xiaorong Zhang, Wei Qian, Daijun Zhou, Xunzhou Yu, Rixing Zhan, Ying Wang, Jun Wu, Weifeng He, Gaoxing Luo

**Affiliations:** ^1^State Key Laboratory of Trauma, Burn and Combined Injury, Institute of Burn Research, Southwest Hospital, Third Military Medical University, Chongqing, China; ^2^Department of Burns, The First Affiliated Hospital of Sun Yat-sen University, Guangzhou, China

**Keywords:** wound healing, angiogenesis, P311, dermal microvascular endothelial cells, matrigel plug assay

## Abstract

P311 was identified to markedly promote cutaneous wound healing by our group. Angiogenesis plays a key role in wound healing. In this study, we sought to define the role of P311 in skin wound angiogenesis. It was noted that P311 was expressed in endothelial cells in the dermis of murine and human skin wounds. The expression of P311 was confirmed in cultured murine dermal microvascular endothelial cells (mDMECs). Moreover, it was found that knockout of P311 could attenuate the formation of tubes and motility of mDMECs significantly *in vitro*. In the subcutaneous Matrigel implant model, the angiogenesis was reduced significantly in P311 knockout mice. In addition, wound healing was delayed in P311 knockout mice compared with that in the wild type. Granulation tissue formation during the defective wound healing showed thinner and blood vessel numbers in wound areas in P311 knockout mice were decreased significantly. A reduction in VEGF and TGFβ1 was also found in P311 KO mice wounds, which implied that P311 may modulate the exprssion of VEGF and TGFβ1 in wound healing. Together, our findings suggest that P311 plays an important role in angiogenesis in wound healing.

## Introduction

P311 (also known as PTZ17, Neuronal protein 3.1) is a highly conserved 8-kDa intracellular protein containing 68 amino acids (Yao et al., 2017). Following firstly identified to expressed in the embryonic brain in mice by Studler (Studler et al., 1993), P311 was found to express in almost all kinds of cells, like motoneurons (Fujitani et al., 2004), kidney tubular epithelial cells (Yao et al., 2015), glioblastomas (McDonough et al., 2005), epidermal stem cells (Li et al., 2016), fibroblast (Tan et al., 2010; Cheng et al., 2017), smooth muscle cells (Badri et al., 2013), which implied wide biological functions of P311. Actually, P311 has been shown to promote the nerve (Fujitani et al., 2004) and lung regeneration (Zhao et al., 2006), glioma invasion (McDonough et al., 2005), and to induce myofibroblast differentiation (Pan et al., 2002; Tan et al., 2010; Li et al., 2016), cell migration (McDonough et al., 2005; Yao et al., 2017). Moreover, a decreased vascular smooth muscle cell contractility, hypotonic blood vessels, and vascular hypotension was found in P311 knockout mice compared with the wild type (Badri et al., 2013), and even a changed behavioral responses in learning and memory (Taylor et al., 2008). However, the molecular mechanisms of P311 biological function have remained unknown. Once the PEST domain (rich in Pro, Glu, Ser, and Thr) in the N-terminus of P311 was thought to be the functional motif that might be the mechanism to indicate function, as the domain also found in short-lived proteins such as transcription factors, cytokines, and signal molecules (Sommer and Wolf, 2014; Varshavsky, 2014). More recently, we found that P311 could increased the activity of Rho A and Rac1 (Yao et al., 2017), which are critical signal transducers for inducing the formation of lamellipodia, filopodia, invadopodia, and blebs during cell migration (Sadok and Marshall, 2014). In addition, P311 was identified as a inducer of EpMyT $(Epidermal stem cell transdifferentiate into myofibroblasts) through TGFβ1/Smad signaling (Li et al., 2016).

Angiogenesis, the process by which new blood vessels are established from preexisting ones (Carmeliet, 2005), is critical to wound healing (Johnson and Wilgus, 2014). Microvascular endothelial cells are the principal parenchymal cells participating in wound angiogenesis (Tonnesen et al., 2000) that involves a phenotypic alteration of endothelial cells, directed migration, and various mitogenic stimuli (Li et al., 2003). Our previous studies demonstrated that P311 was a crucial factor in wound healing (Li et al., 2016; Yao et al., 2017). However, the possible role of P311 in cutaneous wounds angiogenesis was still unknown.

Here we stated that in skin wound healing P311 deficiency resulted in an attenuated angiogenesis. Immunofluorescence results manifested that dermal microvascular endothelial cells expressed protein P311 and highly expressed it under the injury condition, indicating a potential role for P311 in angiogenesis. We then found that a deficiency of P311 decreased the function of endothelial cells (ECs) *in vitro* and angiogenesis in subcutaneous Matrigel plugs *in vivo*. In addition, in full-thickness excisional skin wounds, we observed that P311 knockout leaded a weakened angiogenic response and tissue repair.

## Materials and methods

### Human skin wound tissues

Human skin wound specimens were obtained from 4 patients whose limbs or trunks suffered injury from flame or boiling water with the patient's consent. Ethics approval was granted by the Medical and Ethical Committees of the Southwest Hospital, the Third Military Medical University.

### Mice

The P311 knockout (KO) mice were created, characterized, and genotyped as described previously (Taylor et al., 2008) and kindly gifted by Prof. Gregory A Taylor. All the mice were maintained in the Animal Institutes of Daping Hospital, the Third Military Medical University, with the animal license SCXK(J)2007-017. All protocols involving animals were considered and approved by the Southwestern Hospital Institutional Review Board.

### Culture and characterization of mDMECs

Murine dermal microvascular endothelial cells (mDMECs) were isolated from 3-day-old mouse skin and purified them using magnetic sorting method as reported before (Talavera-Adame et al., 2011) with some modifications. Briefly, the whole skin sheets were washed twice with sterile PBS, cut into 5 × 5 mm pieces, washed again with PBS and digested with 0.5 g/l of Dispase II (04942078001; Roche) at 4°C overnight. Next, the epidermis were kept separate from dermis carefully. The dermal sheets were incubated in 10 ml Medium 199 (M0393, Sigma) containing 40 mg collagenase I (LS004196, Worthington), 0.01% DNase I, and 2% FBS for 45 min at 37°C to release cells. The isolated cells were subjected to purification by magnetic activated cell sorting (MACS) at Passage0 and Passage 2 with CD31 MicroBeads (130-097-418, Miltenyl Biotec). Magnetic sorting was performed by MACS kit according to manufacturer's instructions. The complete medium containing Medium 131, Kit (M131500, Gibco), Microvascular Growth Supplement (MVGS) (S00525, Gibco), 10% fetal bovine serum (FBS) (10099141, Gibco) and 100 U/mL of penicillin and streptomycin (15140122, Gibco) was used to culture the cells. The mDMECs were characterized by CD31 and CD34 as described previously (Cha et al., 2005). FITC-conjugated specific CD31 (11-0311-8, Bioscience) and PE-conjugated specific CD34 (119307, Biolegend) were used to identify the cells with Attune Acoustic Focusing Cytometer (Applied Biosystems, Life Technologies, CA, USA).

### Quantitative real-time PCR

Total RNA was extracted from cells with the RNeasy Mini Kit (QIAGEN, 74104). According to the manufacturer's instructions, we synthesized the cDNA with a cDNA Synthesis Kit (TOYOBO, FSK-100). SYBR Green Master Mix (Toyobo, QPK-201) was used to perform the Real-time PCR on 7500 Real Time PCR System (Applied Biosystems Instruments) with the following primer: P311, 5′-GAGGCTTCCTAAGGGAAGACTT-3′ and 5′-AAGTGGAGGTAAC TGATTCTTGG-3′; GAPDH, 5′-CGTGCCGCCTGGAGAAAC-3′ and 5′-AGTGGGAGTTGCTGTTGAAGTC-3′.

### Tube formation assay *in vitro*

As described previously (DeCicco-Skinner et al., 2014), 2 × 10^4^ mDMECs were seeded into each well of 96-well plate, which was coated with Matrigel. Eight hours later, the tube formation was photographed. The number of nobes and length of tubes was measured by the ImageJ 1.48V software (NIH, USA). Each group has six replicates in one experiment. The experiment was repeated for three times.

### Scratch wound migration assay

As described previously (Yao et al., 2017), the 2 × 10^4^ mDMECs were seeded into each well of 24-well plates with complete medium and cultured to reach confluence. The scratching wounds were created in the monolayer (0 h) with the pipette tips and then monitored for 24 h using a Zeiss video microscope. Measurements were performed using ImageJ 1.48V software (NIH, USA). Each group has six replicates in one experiment. The experiment was repeated for three times.

### Proliferation assays

Proliferation assays were performed using CCK8 reagent (CK04, Dojindo). 3 × 10^3^ mDMECs were seeded into each well of 96-well plate with complete medium. The absorbance was measured at 450 nm on 1, 3, 5, 7 d after culturing the cells. Each group has six replicates in one experiment. The experiment was repeated for three times.

### Cell cycle analysis

According to the manufacturer of Cell cycle kit (GC001, G.fan), the cells were fixed, washed, stained for propidium iodide (PI) solution containing 200 mg/ml RNase A and 0.1% Triton-X-100. We used Attune Acoustic Focusing Cytometer (Applied Biosystems, Life Technologies, CA, USA) to analyze the prepared cells, and then the data were analyzed using FlowJo software (Tree Star Incorporation, USA). Each group has five replicates in one experiment. The experiment was repeated for three times.

### Matrigel plug assay

Matrigel plug assay was performed as described before (Herkenne et al., 2015). Briefly, 500 μl Matrigel (BD Biosciences) supplemented with VEGF (500 ng/ml), and heparin (0.0025 U/ml) was injected subcutaneously into the right or left flanks of the mice. Seven days later, the matrigel were harvested, fixed with 4% paraformaldehyde, embedded in parafin and sectioned. The sections were stained with H&E and examined under a light microscope (LEICA, Germany, CTR6000). The number of endothelial cell nuclei was counted in 5–10 no-overlapping visual fields.

### Wound-healing assays

The male and age-matched (12-week-old) mice were selected from the P311 WT and KO mice. The full-thickness excisional skin wound model was created as described previously (Xu et al., 2015). Briefly before the surgery, the hair of the dorsal surface of mice was shaved and cleaned. During anesthetized with 1% pentobarbital via intraperitoneal injection (0.01 mg/g of body weight), two full-thickness excisional skin wounds were made on the dorsal surface with a 4-mm round skin biopsy punch. On day 0, day 3, day 5, day 7, the wounds were photographed using a digital camera. The wounds were measured by the ImageJ 1.48V software (NIH, USA). The amount of wound (wound area%) was calculated using the following formula:

$$Wound\;area\left(\%\right)=SW_{n}/SW_{0}\times 100\%$$

*SW*_0_ stood for the size of the initial wound area and *SW*_*n*_ represented for the size of wound area on the nth day postsurgery.

### Immunofluorescence and immunohistochemistry staining

The procedure of Immunofluorescence (IF) and Immunohistochemistry (IHC) staining were performed as described previously (Li et al., 2016). Briefly, for the cultured mDMECs, the cells were washed with PBS three times and fixed in 4% paraformaldehyde (PFA) for 20 min at room temperature. Then after another three times in PBS and blocking in 10% donkey serum, the cells were incubated with the primary antibody at 4°C overnight. For the section, the formalin-fixed and paraffin-embedded samples were sectioned and mounted on polylysine-coated slides. Followed by deparaffinized and rehydrated, the sections were incubated in boiling in 10 mM citrate buffers (pH 6.0) for 15 min to unmask the antigen. Then after blocking, the sections were incubated with the primary antibody as indicated above. For the IF, the secondary antibody conjugated with Alexa Fluor 488 or CY3 was used to visualize the signals. Avidin peroxidase reagent (SP-9001, Zhongshan Biology Company) coordinating with 3,30-diaminobenzidine tetrahydrochloride (DAB) chromogenic agent (ZLI-9017, Zhongshan Biology Company) was used to visualize the signals in IHC. The primary antibodies were listed: CD31 (1:100, Abcam, ab28364,); vWF (1:200, Abcam, ab11713); P311(1:100, Novus, NBP1-84315); TGF-β1(1:200, Novus, NB100-91995) and VEGF (1:100, Abcam, AB46154). The sections were reviewed by a light microscope (LEICA, Germany, CTR6000).

### Hematoxylin-eosin (H&E) staining and analysis

The mice were sacrificed on 3rd and 5th day after wounding and then the samples were fixed with 4% paraformaldehyde, embedded in paraffin, sectioned and mounted on slides. For quantification, sections were stained with HE and photographed. Measurements were performed using ImageJ 1.48V software (NIH, USA).

### Western blots

The procedure was performed as described previously (Cheng et al., 2017). Briefly, after grounding in liquid nitrogen, homogenizing with the whole Cell Lysis Kit (Keygen, KGP2100) and centrifuging at 12,000 g for 15 min, The supernatants were collected from the skin wounds. The protein concentrations were determined by BCA Assay (Pierce, 23225). Forty milligrams of proteins for each sample were separated on 10% SDS–PAGE gel, and then transferred electrophoretically to polyvinylidene difluoride (PVDF) (Millipore) membranes. After blocked with 3% bovine serum albumin (BSA), incubated with primary antibody at 4°C overnight and incubated with horseradish peroxidase-conjugated secondary antibodies at room temperature for 1 h, the Molecular Imager ChemiDoc TMXRS+ Imaging System (BioRad) and an enhanced chemiluminescence (ECL) detection kit (Pierce, 35055) were cooperated to detect the signal. The primary antibodies were as follows: CD31 (1:500, Abcam, ab28364,); VEGF (1:1000, Abcam, AB46154); TGF-β1(1:500, Novus, NB100-91995).

### Measurement of VEGF and TGF-β1 concentrations in the wounds by ELISA

After homogenizing, the acidified lysates from normal skin, healing wounds were centrifuged at 12,000 g for 15 min at 4°C. The supernatants were used to measure the VEGF and TGF-β1 concentrations by ELISA. According to the manufacturer's instructions, the assays for VEGF and TGF-β1 were performed with the ELISA (enzyme-linked immunosorbent assay) kits (Abcam, ab100752) and (R&D Systems, DY1679-05), respectively.

### Statistical analysis

The data were presented as mean ± *SD* (standard deviation) and SPSS 18.0 software was used to analyse the data with unpaired, two-tailed Student's *T*-test. *P* < 0.05 was considered statistically significant.

## Results

### Localization of P311 in dermis

**Figure 1:**
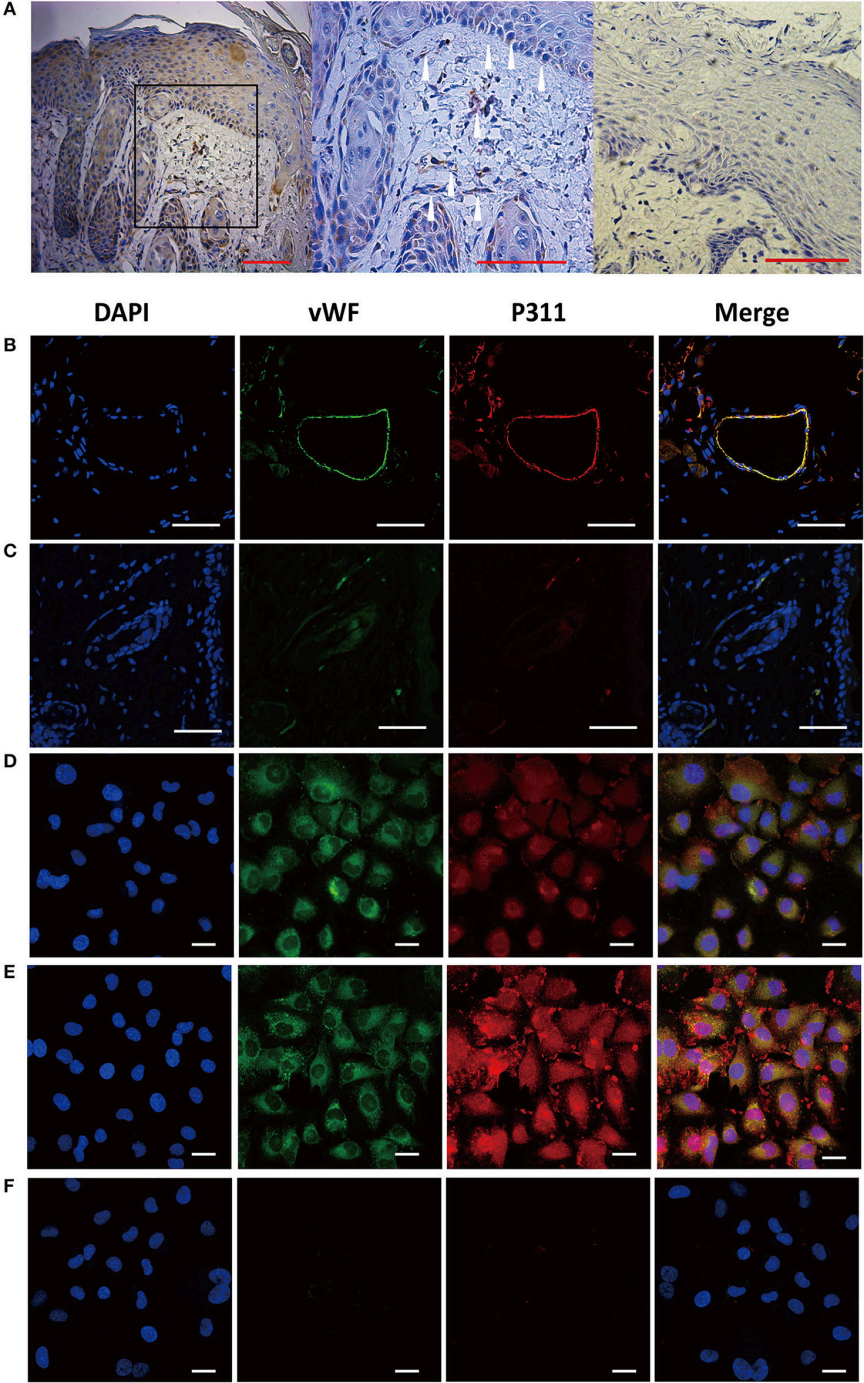
Expression of P311 in dermis and dermal microvascular cells. **(A)** Representative IHC stains for P311 in dermis of human skin wounds. Positivity is represented by brown color. The white arrowheads, P311^+^ cells. Negative control without primary antibody was shown in the right panel. Scar bar = 100 μm. **(B)** Localization of P311 in relationship to ECs (vWF+) in mouse dermis. Scar bar = 100 μm. **(C)** Negative control without primary antibody was shown compared with **(B)**. Immunoflorescence for vWF and P311 was performed in mouse dermal microvascular cells **(D)** and the cells stimulating by IL1β (a common injury signals) **(E)** for 48 h. **(F)** Negative control without primary antibody was shown compared with **(D,E)**. Nuclei were stained by DAPI in blue, vWF was labeled by AF488 in green, and P311 was labeled by AF594 in red. Scar bar = 25 μm.

Previous study has reported that P311 expression was up-regulated in skin wounds and scars (Cheng et al., 2017), while little P311 protein was detected in normal skin (Cheng et al., 2017; Yao et al., 2017). To localize P311 in skin, the samples from the human skin wounds were selected and immunohistochemistry staining of vertical sections of the samples was performed. Expression of P311 was found in epidermal stem cells in epidermis and fibroblast was also found to express P311 in dermis (Figure 1A), which was consistent with our previous studies (Li et al., 2016; Cheng et al., 2017; Yao et al., 2017). Intriguingly, we found some P311^+^ cells in the blood tube-like structures in human skin wounds (Figure 1A), which implied that the vascular endothelial cells might express P311. To further confirm, immunofluorescence staining of sections of skin wounds from P311wild type (WT) mice was performed. The vWF^+^ ECs were found to express P311 in the granulation tissue (Figure 1B), which meant the vascular endothelial cells expressed P311.

### Expression of P311 in mDMECs

To clarify the expression of P311 in vascular endothelial cells, immunofluorescence staining was performed to detect the expression of P311 in murine dermal microvascular endothelial cells (mDMECs), the vWF^+^ cells. mDMECs were isolated from the skin of 3-day-old P311 WT mice by magnetic separation with CD31 MicroBeads. As shown in Supplementary Figure 1A, characteristic cobble-stone morphology of confluent endothelial cells (ECs) was observed. Approximately 89.2% of cultured cells were CD31^+^ cells and 90.7% of cultured cells were CD34^+^ cells, which are widely characterized as endothelial cells (Supplementary Figure 1B). Immunofluorescence result showed that almost all the cultured cells expressed vWF and P311 (Figure 1D); after a 48-h stimulation of IL1β (a common injury signal), the vWF^+^ cells highly expressed P311 (Figure 1E). Real-time quantitative PCR (qPCR) studies demonstrated that P311 mRNA expression was increased after a 48-h stimulation of IL1β (Supplementary Figure 2).

Taken together, these results showed that dermal microvascular endothelial cells expressed protein P311 and highly expressed it under the injury condition.

### P311 deficiency impaires endothelial cell migration and tube formation *in vitro*

**Figure 2:**
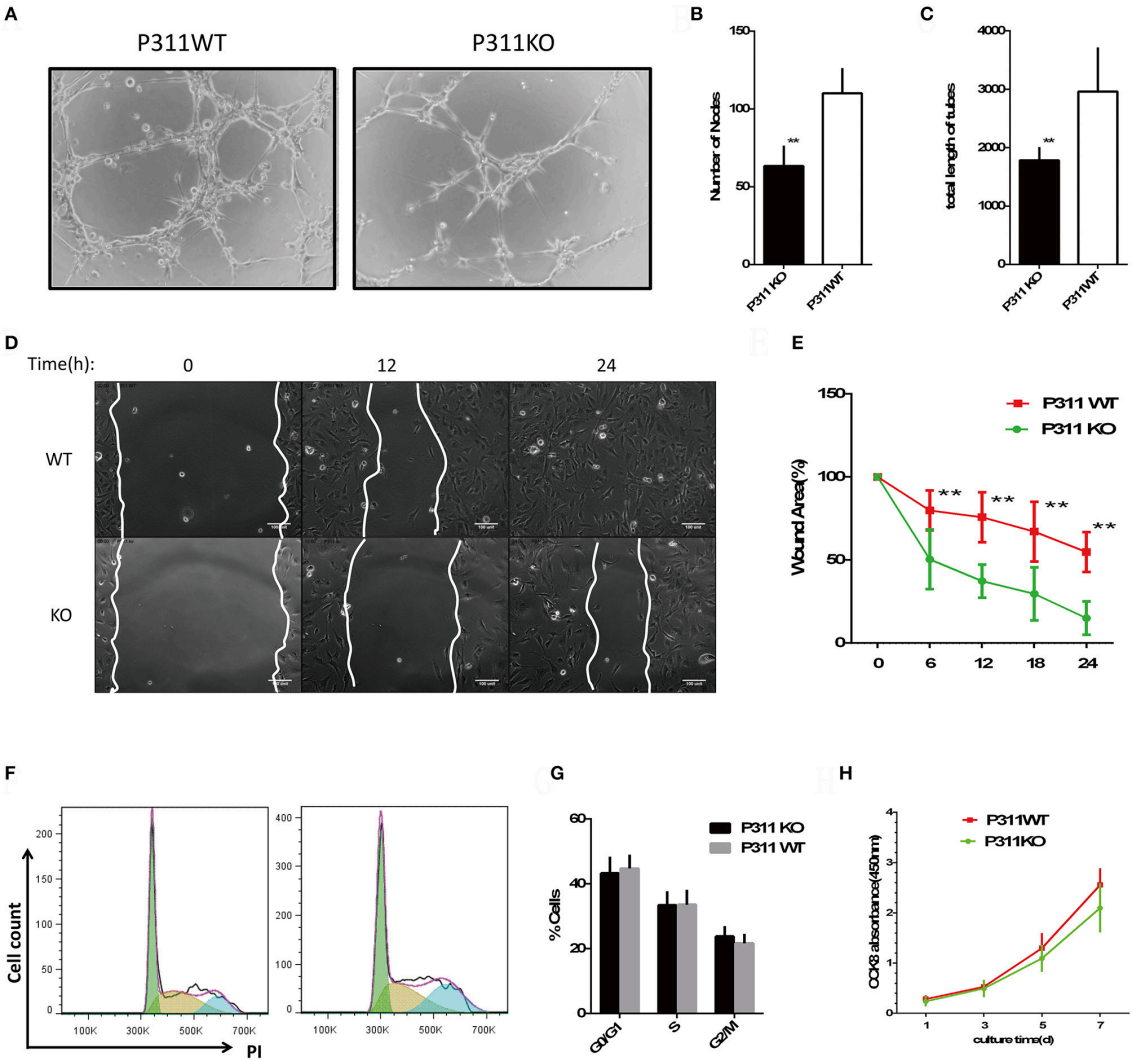
Effect of P311 deficiency on endothelial cell function *in vitro*. **(A)** Representative images of tube formation in Matrigel. Scar bar = 100 μm. **(B,C)** Quantitation of tube formation (*n* = 6 per group). **(B)** Number of nobes. **(C)** Total length of tubes. **(D)** Representative images of recovery of scratched areas by cell migration at 12 and 24 h (*n* = 6 per group). The white line stands for the leading edge of migration cells. Scale bars = 100 μm. **(E)** Quantitation of the wound area (*n* = 6 per group). **(F)** Representative flow cytometry cell cycle of PI. **(G)** Quantitation data of flow cytometry cell cycle (*n* = 5 per group). **(H)** CCK8 assay was performed to assess the effect of P311 on the proliferation (*n* = 6 per group). Data were expressed as means ± *SD*. ^**^P < 0.01, P311 WT vs. KO. All experiments were repeated for three times with reproducible results.

The dermal microvascular endothelium plays a central role in angiogenesis during skin wound healing. So we isolated mDMECs to assess the potential influence of P311 on ECs function *in vitro*. No difference was observed in the morphology and purity of the mDMECs isolated from P311 WT and P311 KO mice (Supplementary Figure 1A). The P311KO mDMECs showed an impaired ability of tube formation (Figures 2A,B). In matrigel assays, which allow determination of the ECs potential to give rise to blood vessel-like tubular structures *in vitro*, thus mimicking angiogenesis, 8 h after seeding P311KO mDMECs on Matrigel, the number of nodes was significantly less (*P* < 0.01) and total length of tubes was significantly shorter (*P* < 0.01) compared with the P311 WT mDMECs.

ECs migration is indispensable for neovessel formation. We examined the effect of P311 on ECs migratory capacity. As shown in Figures 2C,D and Supplementary S1, P311 deficiency impaired mDMECs migration *in vitro*. In wound scratch assays, time-lapse microscopy was used to measure the migration rate of mDMECs into a denuded area over a 24-h period. The entire denuded area was almost filled by P31 WT mDMECs within 24 h, whereas P311 KO mDMECs had covered the area only partially.

Finally, we tested the effect of P311 on ECs proliferation, which is intrinsic to the angiogenesis process. Flow cytometry was utilized to analyze the impact of P311 on mDMECs cycle progression. In almost the same way, both kinds of mDMECs entered the cell cycle and progressed through S-phase and G2-M (Figures 2F,G). Meanwhile, CCK8 assay was performed after the cells were cultured for 1, 3, 5, 7 d, and no significant difference was observed at any time point between the P311 WT and P311 KO mDMECs.

Together, P311 deficiency mainly impaired the abilities of mDMECs tube formation and migration *in vitro*.

### Role of P311 for angiogenesis *in vivo*

**Figure 3:**
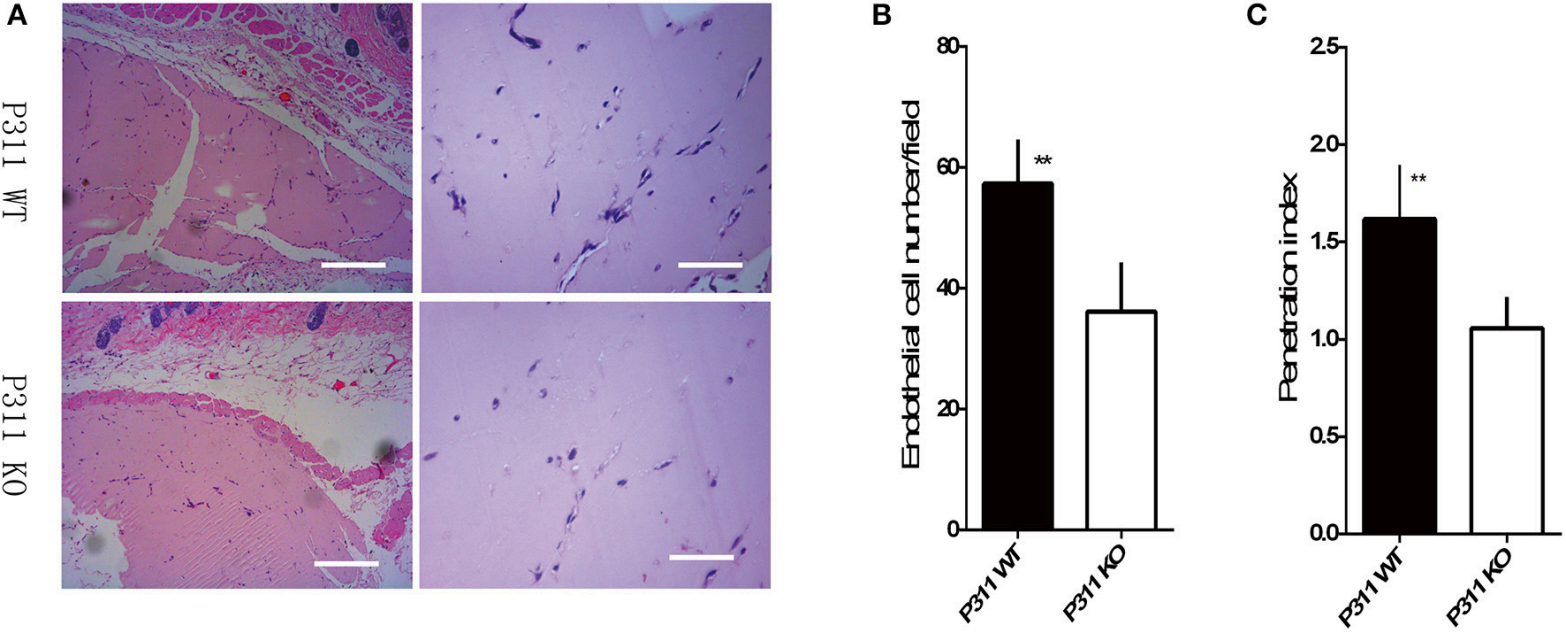
Effect of P311 on angiogenesis in subcutaneous Matrigel plugs. **(A)** Representative cross-sections of H&E-stained of the Matrigel plugs, harvested on the 7th day after implantation into P311 WT and P311 KO mice. Scale bars = 200, 50 μm. **(B,C)** Quantitation of neoangiogenesis. **(B)** Number of endothelial cell nuclei in the matrigel implants (*n* = 4 per group). **(C)** Penetration Index (Penetration of new vessels into the implants, penetration index = area of vessels/overall Matrigel area) (*n* = 4 per group). Data were expressed as means ± *SD*. ^**^P < 0.01, P311 WT vs. KO. The experiment was repeated for three times with reproducible results.

To further determine whether P311 knockout also affects angiogenesis *in vivo*, we performed matrigel plug assays in P311 WT and P311 KO mice. As shown in Figure 3, the number of endothelial cell, which invaded into the plugs of P311 KO mice, was significantly lower compared with that of P311WT mice. Thus, the above data indicate that P311 shows a pro-angiogenic effects *in vitro* and *in vivo*.

### Delayed wound healing in P311 KO mice

Angiogenesis, the process by which new blood vessels are formed from preexisting ones, plays an essential role in tissue regeneration. Based on observations above, we hypothesized that P311 might impact tissue regeneration processes through modifying the angiogenesis and we studied the process of full-thickness skin wounds in P311 WT and P311 KO mice to test this hypothesis.

#### Macroscopic analysis

**Figure 4:**
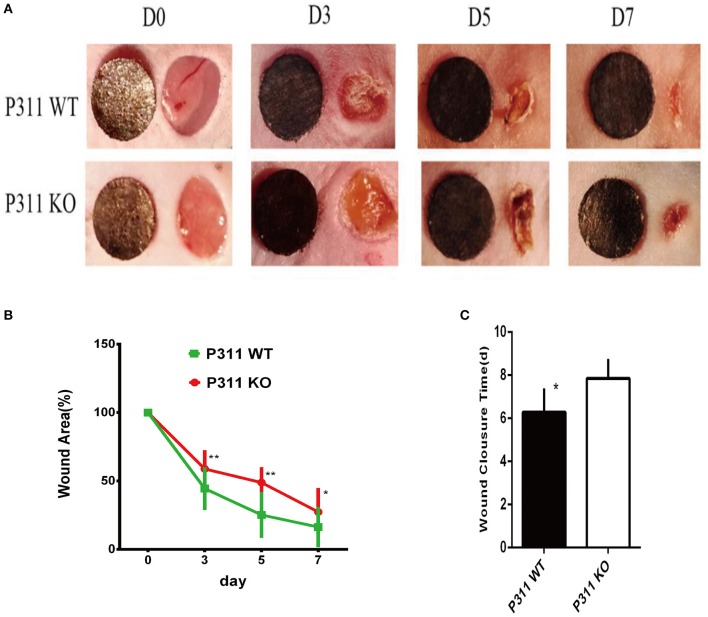
Delayed wound healing in P311-null mice. **(A)** The macroscopic appearance of the wounds post-surgery at different time-points. **(B)** The amounts of wound healing at different time (*n* = 6 per group). **(C)** Wound closure times (*n* = 6 per group). Data were expressed as means ± *SD*. ^*^P < 0.05, ^**^P < 0.01, P311 WT vs. KO. The experiment was repeated for three times with reproducible results.

Figure 4 showed the macroscopic analysis of the wound closure. We found that on the 3rd day after wounding to the wound closure, skin wounds in P311 KO mice maintained a macroscopic greater wound opening than that in P311 WT mice. Quantification of the digitized images of the wound areas revealed that on 7th day post-wound, only ~16.58% of the wound area was left in P311 WT mice, but almost 38.82% wound area was still open in P311 KO mice (P311^+/+^, 27.16% vs. P311^−/−^, 49.48%, *P* < 0.05, on 5th day; P311^+/+^, 52.49% vs. P311^−/−^, 65.51%, *P* < 0.05, on 3th day). Meanwhile, P311 deficiency prolonged wound closure time (Figure 4C). The average wound closure time in P311 WT mice and P311 KO were 6.2 and 7.8 days, respectively.

#### Microscopic analysis

**Figure 5:**
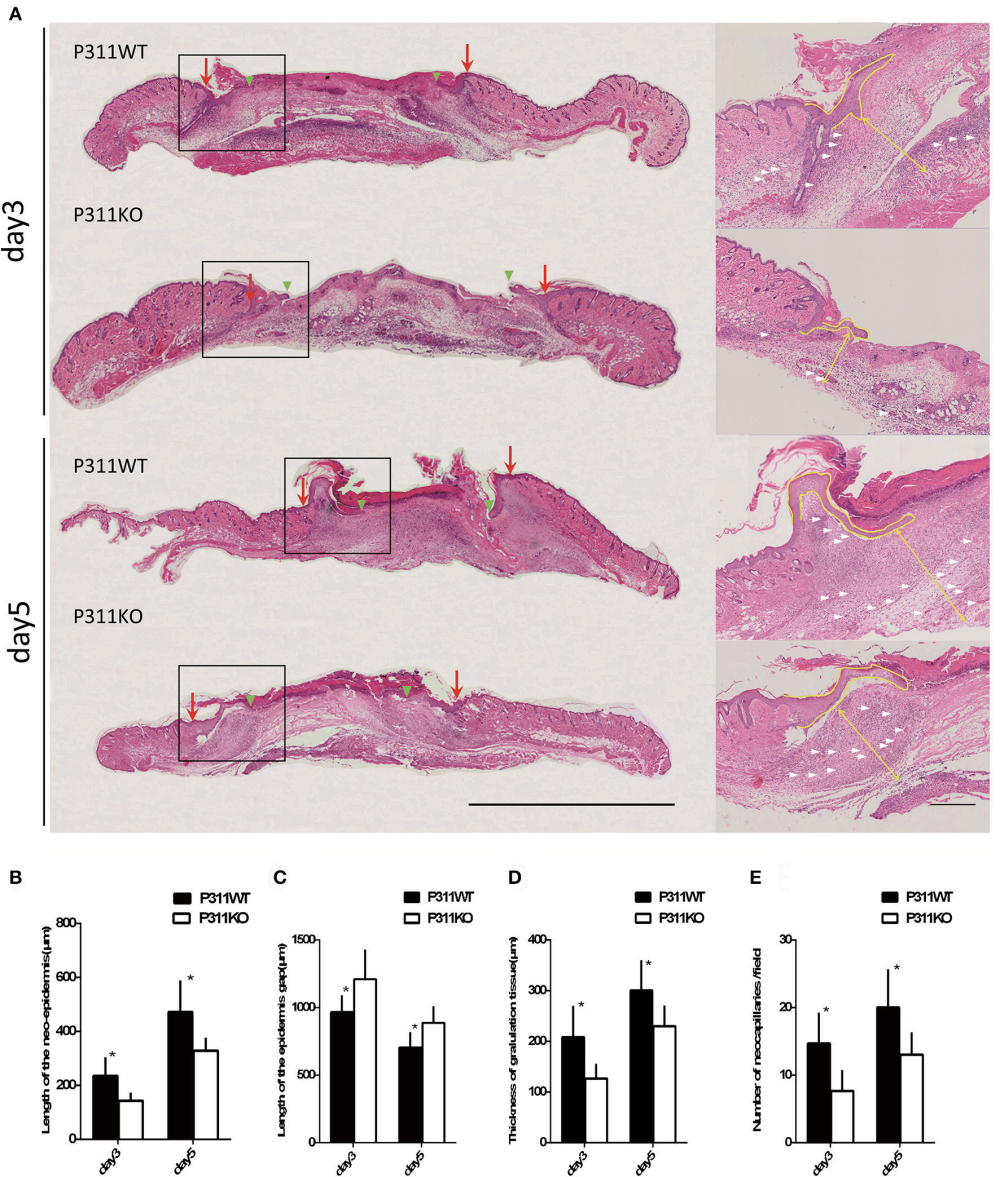
Effect of P311 expression on wound re-epithelialization and granulation tissue. **(A)** H&E-stained sections of excisional skin wound samples from P311 WT and P311 KO mice on 3rd day and 5th day after wounding. Scale bars = 1,000, 200 μm. s, scab; g, granulation tissue; d, dermis; ne, neo-epithelium; pc, panniculus carnosus. Red arrows indicate wound edges; green arrowheads, tips of epithelial tongues; white arrows, microvessels. **(B)** Quantitation of the neo-epidermal length (*n* = 6 per group). **(C)** Quantitation of the wound width (*n* = 6 per group). **(D)** Quantitation of the thickness of granulation tissue (*n* = 6 per group). **(E)** Quantitation of the neocapillaries (*n* = 6 per group). Data were expressed as means ± *SD*. ^*^P < 0.05, P311 WT vs. KO. The experiment was repeated for three times with reproducible results.

On the HE staining sections, Measurement of the granulation tissue thickness indicated that P311 deficiency reduced granulation tissue remodeling, as the average thickness was thinner in P311 KO mice than that in P311 WT mice (P311^+/+^, 300 μm vs. P311^−/−^, 229.5 μm, *P* < 0.05, on 5th day; P311^+/+^, 208.167 μm vs. P311^−/−^, 126.67 μm, *P* < 0.05, on 3rd day; Figures 5A,D,E). Comparing the average number of neocapillaries in the wound, significant difference occurred between the two groups (P311^+/+^, 20 vs. P311^−/−^, 13, *P* < 0.05, on 5th day; P311^+/+^, 14.67 vs. P311^−/−^, 7.67, *P* < 0.05, on 3rd day). We also found that P311 knockout delayed the wound re-epithelialization significantly, in consistent with a previous study that identified P311 as a promoter of epidermal stem cell migration (Yao et al., 2017). The average length of the neo-epithelium was significantly shorter (P311^+/+^, 472.17 μm vs. P311^−/−^, 328.17 μm, *P* < 0.05, on 5th day; P311^+/+^, 235.00 μm vs. P311^−/−^, 143.00 μm, *P* < 0.05, on 3th day) and the average gap of the two neoepidermal leading edges was significantly larger (P311^+/+^, 706 μm vs. P311^−/−^, 864 μm, *P* < 0.05, on 5th day; P311^+/+^, 963.67 μm vs. P311^−/−^, 1208.67 μm, *P* < 0.05, on 3th day) in P311^−/−^ mice (Figures 5A–C).

**Figure 6:**
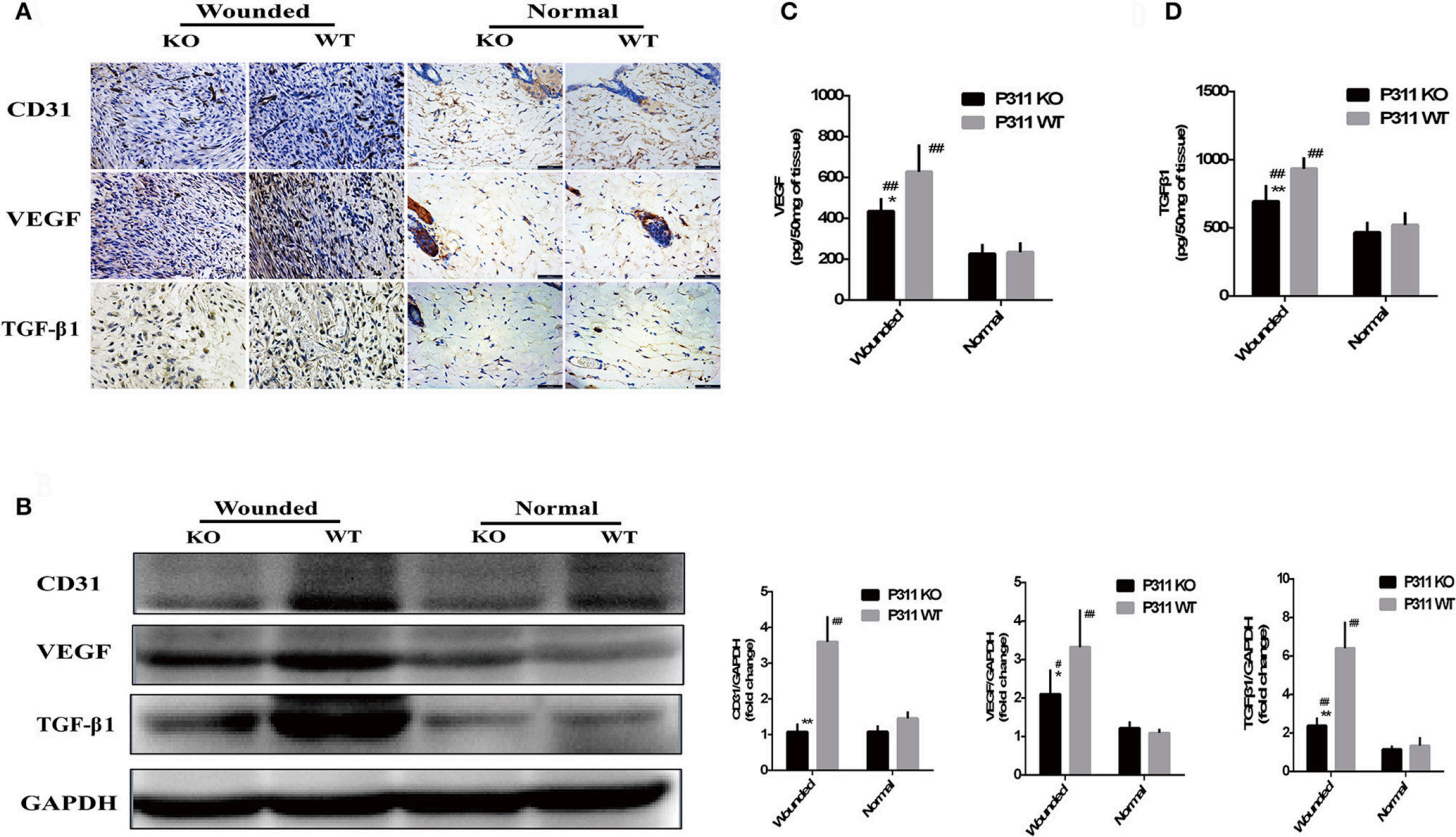
Effect of P311 expression on wound angiogenesis. **(A)** IHC staining of granulation tissues (day 5) for CD31, VEGF, and TGFβ1. **(B)** Western blot showed the expression of CD31, VEGF, and TGFβ1 in each group. Quantitation of the protein levels of CD31, VEGF, and TGFβ1 in each group (day 5). Levels of VEGF **(C)** and TGFβ1 **(D)** releasing during skin wound (day 5) were measured by sandwich ELISA. Data were expressed as means ± *SD*. *n* = 5 per group. ^*^P < 0.05, ^**^P < 0.01, P311 WT vs. KO. ^#^*P* < 0.05, ^##^*P* < 0.01, wounded vs. normal. The experiment was repeated for three times with reproducible results.

On immunohistochemical staining sections, CD31 staining was performed to visible the microvessels in the granulation tissues. Consistent with the observation on HE staining, we observed a significantly less efficient growth of new vessels into the wounds in P311 KO wound mice, compared with P311 WT wound mice, while no difference was noted between the P311 WT normal skin and P311 KO normal skin (Figure 6A). A significant increase in VEGF and TGFβ1 was observed after injury (normal vs. wound), and a reduction in VEGF and TGFβ1 immunoreactivity consistent with the decrease in neoangiogenesis was also observed in P311 KO mice, compared with P311 WT wound mice in the same tissues (Figure 6A). The decreased expression of CD31, VEGF, and TGFβ1 in the samples was further confirmed by Western blotting analysis (Figure 6B). The CD31, VEGF, and TGFβ1 protein levels in P311 WT wounds (D5) increased significantly, compared with P311 KO wounds (*P* < 0.05) and P311 normal skin. The concentration of VEGF and TGFβ1 in supernatants of P311 KO wound mice as detected by ELISA was significantly lower than in that of P311 WT wound mice (Figures 6C,D). Thus, P311 deficiency leads to attenuated angiogenesis in cutaneous wound healing.

## Discussion

Angiogenesis, which is highly regulated by diverse factors in a consecutive, concerted, or synergistic manner (Li et al., 2003), plays a fundamental role in wound healing, tumor growth, invasion, and metastasis (Carmeliet, 2005). More recently, we found that P311 could promote cutaneous wound healing through increasing the activity of RhoA and Rac1 (Yao et al., 2017) and inducing EpMyT (Epidermal stem cell transdifferentiate into myofibroblasts; Li et al., 2016). Hence, we carried out experiments to determine whether P311 may have a function in angiogenesis in wound healing.

Here, for the first time, we demonstrated that P311 could promote angiogenesis in wound healing by changed the endothelial response. This function was supported by the following findings of our present study: (1) P311 was colocalized with vWF^+^ ECs in murine dermis and dermal microvascular endothelial cells (mDMECs) expressed protein P311 and highly expressed it under the injury condition. (2) P311 deficiency mDMECs showed decreased abilities in tube formation and migration *in vitro*. (3) *In vivo*, compared with the P311 WT mice, a significantly less number of ECs was detected in subcutaneous Matrigel implant in P311 KO mice. (4) P311 KO mice showed impaired granulation tissue formation and less CD31^+^ ECs in the granulation tissues in wound healing.

Microvascular endothelial cells are the principal parenchymal cells involved in wound angiogenesis (Li et al., 2003). However, no experiments were performed to define whether the microvascular endothelial cells express P311. Using immunofluorescence, we determined that P311 colocalized with vWF^+^ ECs in murine dermis. Further the expression of vWF and P311 in isolated murine dermal microvascular endothelial cells (mDMECs) at steady state and injury condition was detected. These results demonstrated that mDMECs expressed protein P311 and highly expressed it under the injury condition *in vivo* and *in vitro*. Previous studies have showed that P311 expression was little in cells from normal tissue and was obviously increased in cells from injured tissue, implying that P311 might be an injury-dependent protein (Cheng et al., 2017; Yao et al., 2017). Then we examined the influence of P311 deficiency on ECs function *in vitro* using the isolated mDMECs. In consistent with results reported before (Shi et al., 2006; Yao et al., 2017), P311 deficiency mDMECs showed a decreased migration ability. Meantime, the formation of tubes in P311 deficiency mDMECs was also reduced. However, the cell proliferation was almost the same in both mDMECs. In the subcutaneous Matrigel implant, a significant decrease of neoangiogenesis was observed in P311 KO mice. Therefore, it is likely that P311 reduced neoangiogenesis by altering endothelial cell response.

To investigate the effect of the P311 on angiogenesis in wound healing, full-thickness excisional skin wound model was created with P311 KO and P311 WT mice. In a macroscopic analysis, wound healing was significantly delayed in P311 KO mice. Moreover, the average wound closure time was even longer in P311 KO mice. Wounds from P311 KO mice displayed a thinner granulation tissue and CD31^+^EC numbers were reduced in granulation tissue in P311 KO mice, which was further confirmed by western blot. The results indicate that P311 may promote wound healing by enhancing angiogenesis in granulation tissue. Together, P311 regulates angiogenesis in granulation tissue by directly altering endothelial cell response.

In addition, a reduction in VEGF and TGFβ1 was also found in P311 KO mice wounds. The two are both the most critical angiogenic factors that modify the vasculogenesis and angiogenesis (Pardali and Ten, 2009; Pardali et al., 2010; Shibuya, 2013). Vascular endothelial growth factor (VEGF) regulates angiogenesis by binding to the tyrosine kinase receptor VEGFR2, inducing the dimerization, phosphorylation of the receptor and the downstream signaling pathways (Adams and Alitalo, 2007; Koch and Claesson-Welsh, 2012). As endothelial cells are able to produce VEGF and express P311, we speculated that P311 might induce the stimulating effects of VEGF, thus leading to altered endothelial cell response, and then modifying angiogenesis. P311-VEGF/VEGFR2-ECs response may be the mechanism under the angiogenesis. Further study is necessary to confirm the hypothesis.

TGF-β signaling pathways also have been strongly proved to be crucial in vasculogenesis and angiogenesis by a series of genetic manipulations of the pathway components. Recently, P311 was identified as a regulator of TGF-βs and a RNA-binding protein to stimulate TGF-βs translation *in vitro* and *in vivo* by Lucia Schuger group and our group (Li et al., 2016; Cheng et al., 2017). So we speculated that in wound healing P311 might modulate angiogenesis by affecting the secretion of TGFβ. Moreover, TGF-β was found to increase the secretion of VEGF (Trompezinski et al., 2000). So P311 also might modulate angiogenesis through P311-TGFβ-VEGF/VEGFR2 signaling pathway. More studies are needed to further confirm the exact mechanism in P311 modulating the angiogenesis in wound healing.

In summary, for the first time, we demonstrated that P311 could promote angiogenesis in wound healing by changed the endothelial response.

## Ethics statement

In the study, all human experiments were carried out in accordance with the recommendations of the Medical and Ethical Committees of the Southwest Hospital, the Third Military Medical University with written informed consent from all subjects. All subjects gave written informed consent in accordance with the Declaration of Helsinki. The protocol was approved by the Medical and Ethical Committees of the Southwest Hospital, the Third Military Medical University. All animal experimental protocols were approved by the Animal Experimental Ethics Committees of the Third Military Medical University and were also performed in accordance with the guidelines of the Third Military Medical University.

## Author contributions

SW, WH, JW, and GL have made substantial contributions to the conception or design of the work. The majority of the experiments were conducted by SW and XZ. WQ, DZ, XY and GL contributed to collection, analysis, interpretation of the data for the study. SW wrote the first draft of the manuscript. Then RZ, YW, JW, WH and GL revised and edited the manuscript critically for important intellectual content. GL was responsible for obtaining funds. All authors read and approved the manuscript.

### Conflict of interest statement

The authors declare that the research was conducted in the absence of any commercial or financial relationships that could be construed as a potential conflict of interest.
