# Supplementary material for: P311 Deficiency Leads to Attenuated Angiogenesis in Cutaneous Wound Healing
Source: Front Physiol. 2017 Dec 6;8:1004. doi: 10.3389/fphys.2017.01004 (PMC5723677; doi:10.3389/fphys.2017.01004)
Supplement: Supplementary file 1 [file DataSheet1.pdf]

Supplementary Material

P311 deficiency leads to attenuated angiogenesis in cutaneous wound healing

Song Wang *

*** Correspondence:** Weifeng He:whe761211@aliyun.com or Gaoxing Luo:logxw@yahoo.com(GL).

1 Supplementary Data

Supplementary S1. P311 deficiency impaired mDMECs migration in vitro. The up panel stands for mDMECs from P311 KO mice, while the down panel represents mDMECs from P311 WT mice.

2 Supplementary Figures

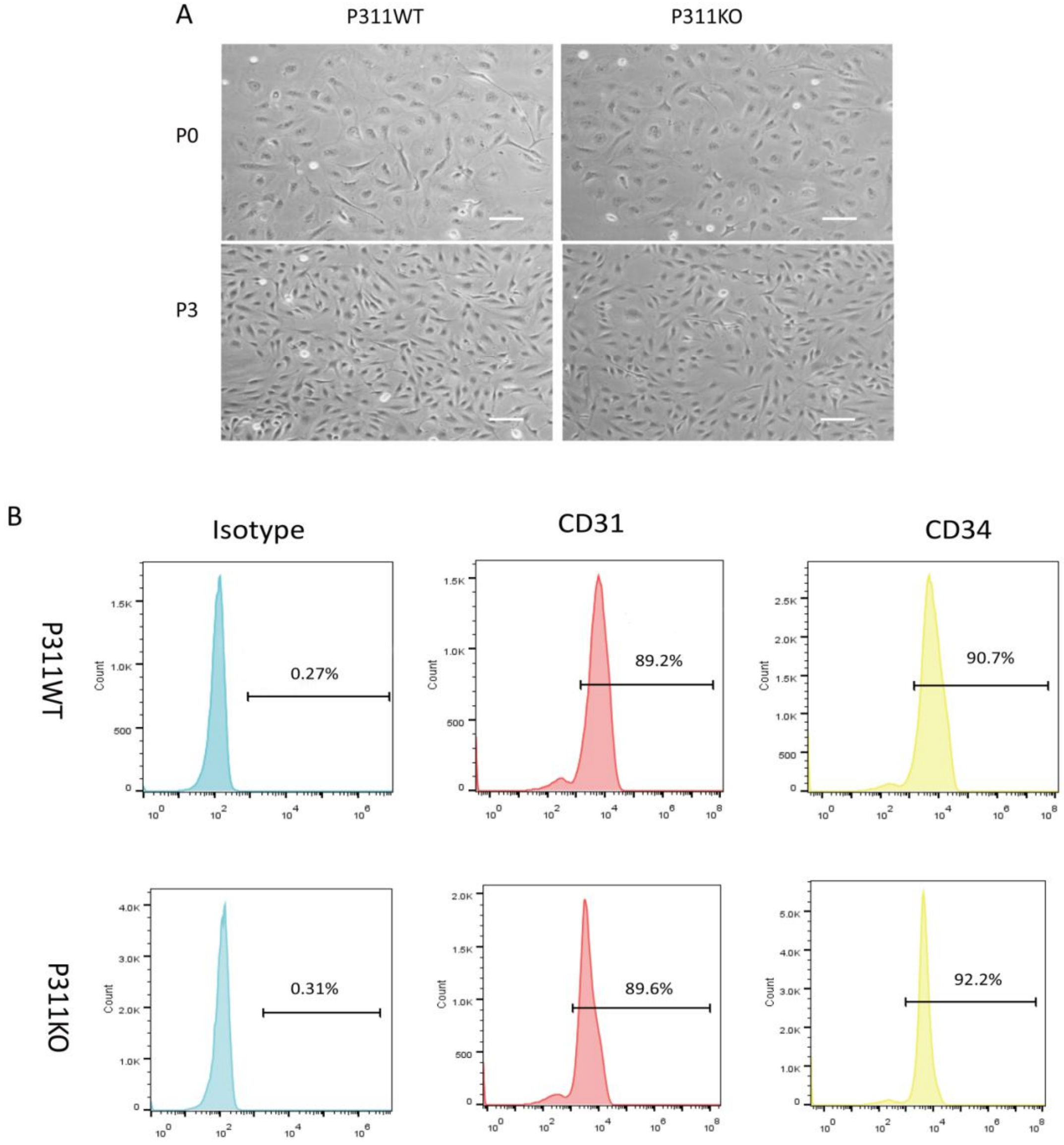

Supplementary Figure 1. The Characterization of cultured murine dermal microvascular endothelial cells (mDMECs).A. The morphology of cultured cells at passage 0 and at passage 3. B. mDMECs at passage 3 express a high level of CD31 and CD34 as shown by flow cytometry analysis. FITC-conjugated specific CD31(11-0311-8,Bioscience) and PE-conjugated specific CD34 (119307,Biolegend) were used.

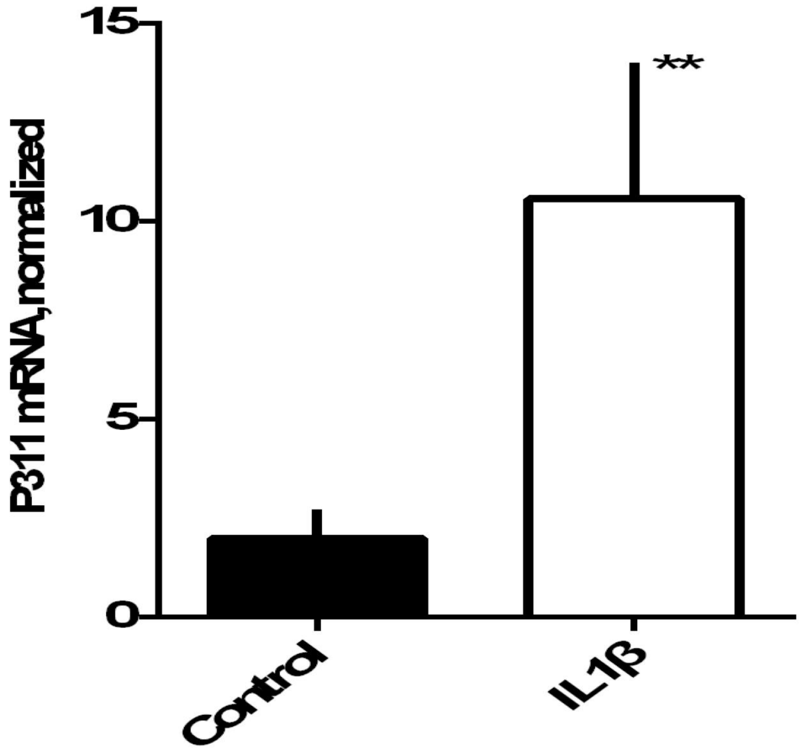

Supplementary Figure 2. P311 was up-regulated by of IL1β(a common injury signal). mDMECs at passage 3 were stimulated with 10ng/ml IL1β for 48 hours. The primers: P311,5'-GAGGCTTCCTAAGGGAAGACTT-3' and 5'-AAGTGGAGGTAAC TGATTCTTGG-3'; GAPDH, 5'-CGTGCCGCCTGGAGAAAC-3' and 5'-AGTGGGAGTTGCTGTTGAAGTC-3'.
